# Prevalence of disability in inflammatory bowel disease: a systematic review and meta-analysis

**DOI:** 10.1093/ibd/izag022

**Published:** 2026-03-05

**Authors:** Olga Maria Nardone, Giulio Calabrese, Alexander C Ford, Fabiana Castiglione, Edoardo Vincenzo Savarino, Vipul Jairath, Yuhong Yuan, Silvio Danese, Tommaso Lorenzo Parigi, Brigida Barberio

**Affiliations:** Department of Public Health, University of Naples Federico II, Naples, Italy; Department of Clinical Medicine and Surgery, University of Naples Federico II, Naples, Italy; Leeds Gastroenterology Institute, St. James’s University Hospital, Leeds, United Kingdom; Leeds Institute of Medical Research at St. James’s, University of Leeds, Leeds, United Kingdom; Department of Clinical Medicine and Surgery, University of Naples Federico II, Naples, Italy; Department of Surgery, Oncology and Gastroenterology, University of Padua, Padua, Italy; Gastroenterology Unit, Azienda Ospedale Università Padova, Padua, Italy; Department of Medicine, Division of Gastroenterology, Western University, London, Ontario, Canada; Alimentiv, Inc, London, Ontario, Canada; London Health Research Institute, London, Ontario, Canada; Department of Epidemiology and Biostatistics, University of Western Ontario, London, Ontario, Canada; Department of Medicine, Division of Gastroenterology, Western University, London, Ontario, Canada; London Health Research Institute, London, Ontario, Canada; Gastroenterology and Endoscopy, IRCCS San Raffaele Hospital and Vita Salute San Raffaele University, Milan, Italy; Gastroenterology and Endoscopy, IRCCS San Raffaele Hospital and Vita Salute San Raffaele University, Milan, Italy; Department of Pathology, Case Western Reserve University, Cleveland, OH, United States; Gastroenterology Unit, Azienda Ospedale Università Padova, Padua, Italy

**Keywords:** inflammatory bowel disease, disability, IBD-DI, IBD-Disk

## Abstract

**Background and aims:**

Disability is a multidimensional concept that includes physical, psychological, and social limitations affecting individuals with inflammatory bowel disease (IBD). Disability is shaped by cultural and health care factors that vary across countries and therefore disability prevalence and characteristics may differ globally. We conducted a systematic review with meta-analysis to assess the pooled prevalence of moderate-to-severe disability and investigate how IBD type, disease activity, geographic location, and questionnaire used influenced prevalence.

**Methods:**

We searched MEDLINE, Embase, and Embase Classic (from database inception to March 1, 2025) for cross-sectional, cohort, registry-based, and case-control studies reporting the prevalence of moderate-to-severe disability based on the IBD Disk or IBD-Disability Index in adults with confirmed IBD.

**Results:**

In total, 17 articles fulfilled the eligibility criteria, including 7897 patients in 17 countries. The pooled prevalence of moderate-to-severe disability in patients with IBD was 29.6% (95% CI, 22.6%-37.1%) and was higher in patients with active IBD (56.9%; 95% CI, 20.3%-89.9%) compared with those with inactive disease (27.0%, 95% CI, 3.3%-62.0%). Based on 3 studies, disease activity increased the odds of moderate-to-severe disability more than 3-fold (odds ratio [OR], 3.13, 95% CI, 1.74-5.64). Stratified by IBD type, moderate-to-severe disability was higher in patients with Crohn disease (36.9%; 95% CI, 25.7%-48.9%) than in ulcerative colitis (30.8%; 95% CI, 19.6%-43.2%), with OR 1.26 (95% CI, 1.06-1.51)

**Conclusions:**

This systematic review is the first, to our knowledge, to show that moderate-to-severe disability affects nearly one-third of patients with IBD, with higher rates in Crohn disease and active disease. Importantly, disability persists in a substantial proportion of patients even during remission, supporting the need for systematic assessment across clinical settings.

## Graphical Abstract

**Graphical Abstract:**
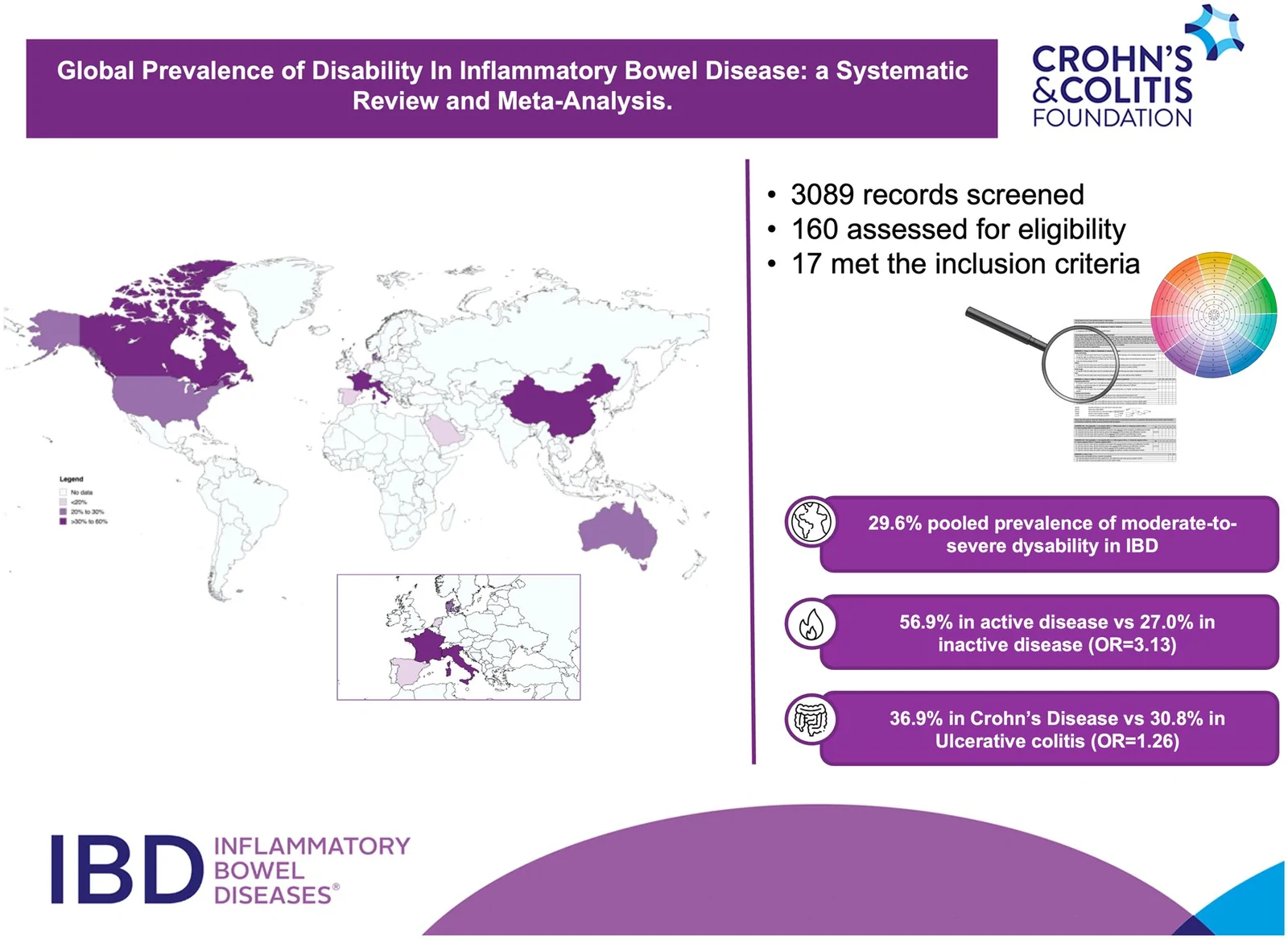

Key Messages *What is already known?*

Preventing disability is increasingly recognized as a long-term endpoint in inflammatory bowel disease management; however, to our knowledge, no previous study has systematically investigated the worldwide prevalence of inflammatory bowel disease–related disability or differences between geographic areas.

*What is new here?*

This i systematic review is the first, to our knowledge, to use validated questionnaires to examine the global prevalence of disability in individuals with inflammatory bowel disease (IBD). Nearly one-third of patients experience moderate-to-severe disability, with higher rates among those with Crohn disease. However, disability can persist even during periods of remission, underscoring the need for systematic disability assessment across all IBD populations.

*How can this study help patient care?*

Quantifying region-level IBD-related disability is the first step in assessing the clinical and psychosocial needs of individuals with IBD, regardless of disease activity, and in planning the resources required to meet future health care demands and promote equity of care.

## Introduction

Inflammatory bowel diseases (IBDs), including ulcerative colitis (UC) and Crohn disease (CD), are lifelong disorders of the gastrointestinal tract characterized by a relapsing–remitting course, which can result in debilitating physical and psychosocial symptoms in patients.^1–5^ Disability is a multidimensional and dynamic construct arising from the interaction between an underlying disease and biological, psychological, and environmental factors that affect an individual’s physical (through impediments to task execution) and mental ability to engage in life activities.^6^^,^^7^^.^^8^ The concept of minimizing IBD-related disability as a long-term treatment endpoint to prevent adverse impacts of this disease on the patient’s life has been proposed in the Selecting Therapeutic Targets in Inflammatory Bowel Disease (STRIDE) initiative STRIDE-II consensus,^9^ and more recently, in the Standard Protocol Items: Recommendations for Interventional Trials (SPIRIT) initiative within the context of disease modification trials.^10^ However, capturing the burden of disability in IBD requires accurate measurement to identify psychosocial determinants and guide targeted strategies for the evolving health care needs of patients.

The most commonly used validated scales to measure disability in adults with IBD are the IBD Disability Index (IBD-DI)^11^^,^^12^ and a self-administered, visually appealing, simplified version of the IBD-DI called the IBD-Disk.^13^ To date, the IBD-Disk has been translated into and validated in at least 12 languages, with the aim of implementing its use in daily clinical practice across different countries.^14–19^ Because the perceptions of disability can be shaped by ethnicity, culture, and social and environmental contexts, it is plausible that the burden of IBD-related disability varies globally. However, to date, no study to our knowledge has systematically investigated the worldwide prevalence of IBD-related disability or whether there are substantial differences in disability between geographic areas.^7^^,^^19–22^ Therefore, we aimed to assess the pooled prevalence of moderate-to-severe disability, measured by either of the 2 validated questionnaires—IBD-Disk and IBD-DI—in adult patients with IBD, and secondarily to explore differences in prevalence rates based on country, economic factors, type of IBD, disease activity, and types of questionnaires.

## Methods

### Search strategy and selection criteria

A comprehensive literature search was conducted in MEDLINE, EMBASE Classic, and EMBASE from database inception until March 1, 2025. Observational studies—consisting of cross-sectional, cohort, registry-based, and case-control designs—were included if they quantified the prevalence of moderate-to-severe disability among adults (aged ≥18 years) with a confirmed diagnosis of IBD. To limit potential prevalence inflation related to small sample sizes, only studies enrolling 30 or more participants were considered eligible. Disability had to be assessed using validated tools, specifically the IBD-DI or the IBD-Disk^11^^,^^13^ (Box 1).

Box 1. Eligibility Criteria.

Cross-sectional surveys or case-control studies.

Adults (>90% of participants aged ≥18 y) with histologically or radiologically confirmed inflammatory bowel disease (Crohn disease, ulcerative colitis).

Participants not specially selected (eg, only those who underwent surgery)

Reported the number (proportion) of patients with moderate-to-severe disability according to a validated questionnaire.

Sample size of  ≥30 participants.

The literature search was performed using a combination of controlled vocabulary terms and free-text keywords related to “ulcerative colitis,” “Crohn disease,” and “inflammatory bowel disease.” These terms were combined using the Boolean operator AND with disability-related keywords, including “disability,” “IBD-DI,” “Inflammatory Bowel Disease Disability Index,” and “IBD-Disk.” No language restrictions were applied. All records identified through the search were initially screened at the title and abstract level for relevance, and full-text articles were subsequently retrieved for detailed eligibility assessment when deemed potentially pertinent.

To identify potentially relevant studies available exclusively as abstracts, major gastroenterology conference proceedings—including those of the American College of Gastroenterology, the European Crohn’s and Colitis Organisation, Digestive Disease Week, and United European Gastroenterology Week—were also screened. In addition, reference lists of all eligible articles were manually reviewed to identify eligible studies. When potentially relevant studies lacked sufficient data for extraction, the corresponding authors were contacted to request additional information, with the aim of maximizing study inclusion. Studies for which the required data could not be obtained and whose authors could not be reached were excluded from the final analysis.

Study eligibility was independently evaluated by 2 reviewers (O.N. and G.C.) using standardized, prespecified eligibility forms. Any discrepancies were adjudicated by a third reviewer (B.B.). Inter-reviewer agreement was quantified using the kappa statistic. Ethical approval was not required for this study. The review protocol was prospectively registered in the PROSPERO database (registration number CRD420251033734), and the conduct and reporting of the review adhered to the Preferred Reporting Items for Systematic Reviews and Meta-Analyses (PRISMA) guidelines^23^ (Table S1).

### Data extraction

Data were extracted independently by 2 investigators (B.B. and G.C.) in a Microsoft Excel spreadsheet (Microsoft 365, Microsoft Corp, Redmond, WA, United States). The following variables were collected for each study: country, method of data collection, type of questionnaire, number of patients with IBD and number of patients with moderate-to severe disability, number of female or male patients, type of IBD (UC, CD, or IBD-unclassified [IBD-U]), number of patients with active or inactive IBD as defined in each study, number of patients with disability, number of patients with active or inactive IBD with moderate-to severe disability, and type of questionnaire used. Moderate-to-severe disability was defined as an overall IBD-Disk score ≥40 or an IBD-DI score^24^ >30.^25^ We focused on moderate-to-severe disability as it is more clinically relevant and likely to have greater impact on patient functional status, health care utilization, and quality of life. Moreover, this threshold reduces the risk of measurement variability and improves comparability across studies.

### Quality assessment

Currently, no universally accepted assessment tool for the quality of cross-sectional studies is available. We used the Newcastle-Ottawa scale, with a total possible score of 9 (higher scores indicating higher quality studies), specifically to evaluate the quality of case-control studies only. Furthermore, risk of bias assessment was performed using the Joanna Briggs Institute (JBI) Critical Appraisal Checklist for Studies Reporting Prevalence Studies.^26^ Each item was scored as 1 for “Yes” and 0 for “No,” “Unclear,” or “Not applicable.” A total quality score percentage (range: 0%-100%) was calculated for each study. Studies scoring 70%-100% were classified as high quality, those scoring 50%-69% points as moderate quality, and those scoring 0%-49% points as low quality, as previously adopted in similar systematic reviews.^27^^,^^28^

### Data analysis

The proportions of patients reporting moderate-to-severe disability were synthesized across studies to estimate an overall pooled prevalence. Prespecified subgroup analyses were conducted according to IBD subtype (UC, CD, or IBD-U), disease activity status, disability assessment tool, and country of origin. Between-group comparisons were expressed as odds ratios (ORs) with 95% CIs.

Statistical heterogeneity across studies was quantified using the *I*^2^ statistic, with values of 25%-49%, 50%-74%, and ≥75% conventionally interpreted as low, moderate, and high heterogeneity, respectively.^29^

Forest plots depicting pooled prevalence estimates and pooled ORs with 95% CIs were generated using R (version 4.5.0) and StatsDirect (version 4.0.4). Where an adequate number of studies (≥10) was available,^31^ potential publication bias was explored by visual inspection of funnel plots and formally tested using Egger’s regression.^30^

To evaluate the potential association between economic factors and disability, we performed a correlation analysis between per capita gross domestic product (GDP) based on purchasing power parity (PPP), expressed in current US dollars from the year of each study’s publication and the reported prevalence of moderate-to-severe disability.^32^

## Results

**Figure 1:**
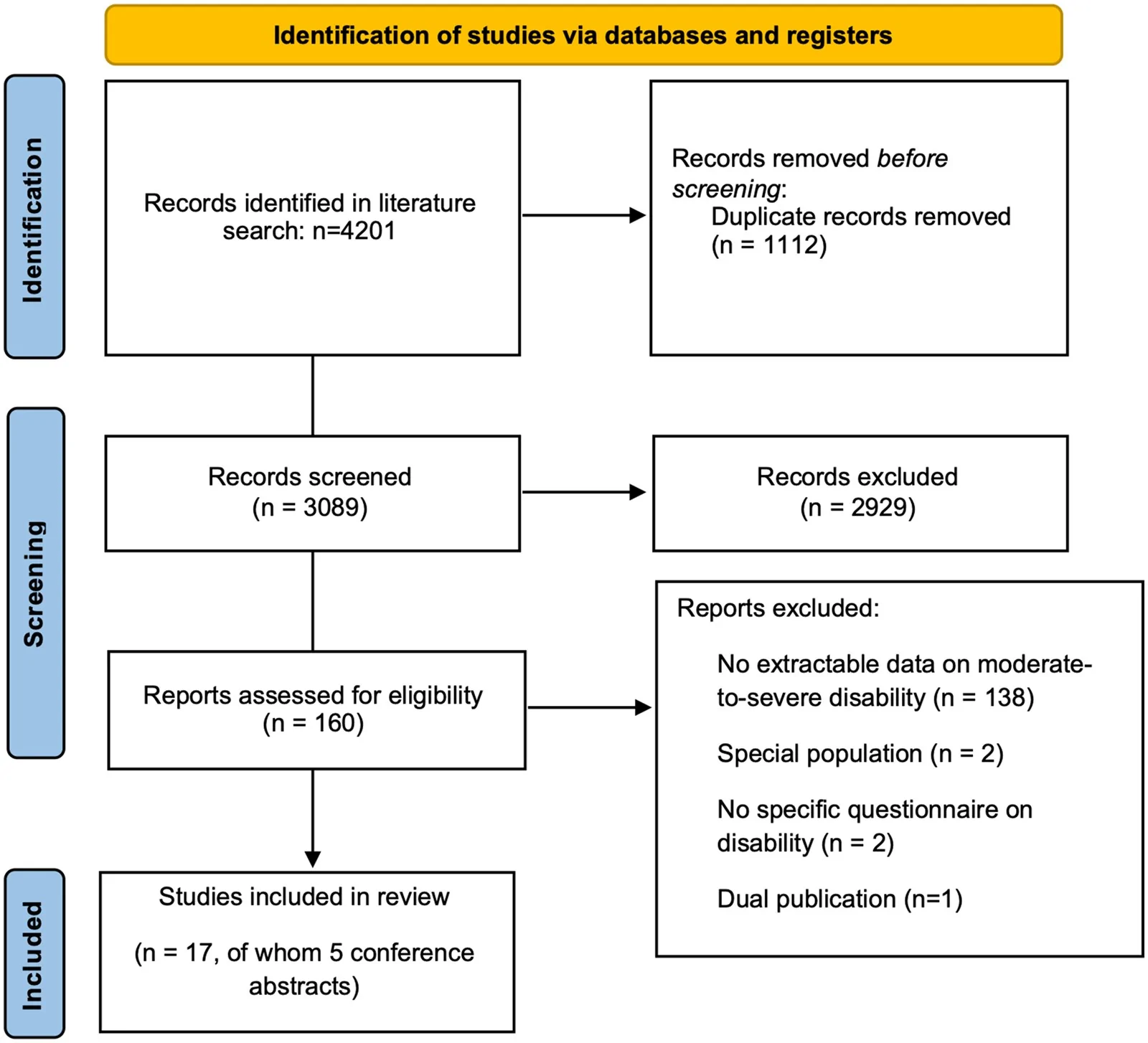
Flow diagram of studies included in the meta-analysis.

The search strategy generated 3089 citations. Of these, 160 articles were identified as potentially relevant to the study question. In total, 17 of these articles fulfilled eligibility criteria (Figure 1), including a total of 7897 patients with IBD recruited from 17 different countries.^7^^,^^10^^,^^14^^,^^20^^,^^21^^,^^25^^,^^33–43^ All studies were conducted in a single country, except one,^39^ which was conducted in multiple European countries, although data for each country were not provided individually.

Among the 17 included studies, 6 studies reported the prevalence of moderate-to-severe disability separately in patients with CD^21^^,^^25^^,^^33^^,^^36^^,^^42^^,^^43^ and UC,^25^^,^^33^^,^^35^^,^^36^^,^^38^^,^^42^ respectively. None of the included studies reported the prevalence of moderate-to-severe disability in patients with IBD-U separately. Only 1 study reported the prevalence of moderate-to-severe disability^36^ in healthy controls, who were included for validation purposes only and not to estimate disability prevalence or enable clinical comparison, as the IBD-DI and IBD-DISK were developed for patients with IBD.

In 1 study^21^ the prevalence of moderate-to-severe disability separately for male and female patients with CD was reported No studies reported sex-specific moderate-to-severe disability prevalence in patients with UC. Therefore, we were unable to calculate the pooled prevalence of moderate-to-severe disability according to sex.

The Newcastle-Ottawa scale was applicable to 1 study,^36^ as it was the sole case-control study included, yielding a score of 7 out of 9. Agreement between investigators on the assessment of study eligibility was good (kappa statistic = 0.8). Detailed characteristics of all included studies are provided in Table 1.

**Table 1:** Characteristics of included studies.

Study 1^st^ Author and reference	Study design	Country	Questionnaire for disability	Disease type	Sample size	Prevalence of moderate-to-severe disability, %	Female sex, %	Age (y) Median (IQR) Mean ± SD	Disease duration (y) Median (IQR) Mean ± SD	Active disease (%)	Biologic therapy (%)
Agrawal^7^	Cross-sectional	United States	IBD-DI	IBD	323	31.0	51.4	–	10 (4-16)	46.4	36.8
Attauabi^33^	Cross-sectional	Denmark	IBD-DI	IBD	336	26.5	–	–	–	–	–
				CD	128	33.6					
				UC	208	22.1					
Bian^21^	Cross-sectional	China	IBD-DI	CD	146	52.1	33.6	31.58 ± 10.00	4.99 ± 4.91	17.8	–
Chao^34^	Cross-sectional	Canada	IBD-DI	IBD	207	30.4	57.5	39 (27‑53)	–	24.2	52.7
Kayal^35^	Cross-sectional	United States	IBD-DI	UC	128	23.4	53.9	38.0 (28.4-54.0)	7.0 (3.0-13.0)	35.9	53.2
Le Berre^19^	Cross-sectional	France	IBD-DISK	IBD	447	31.1	56.8	38.7 ± 13.4	11.3 ± 8.5	29.1	68
Leong^36^	Cross-sectional	Australia	IBD-DI	IBD CD UC	116	21.6	47.4	40.5	8.9	–	33.6
					75	22.7	46.7	39.1	8.7		44.6
					41	19.5	48.8	43.1	9.3		14.6
Lopez-Cortes^37^	Cross-sectional	Spain	IBD-DI	IBD	170	7.1	–	44.31 ± 14.20	–	45.9	–
Meeralam^20^	Cross-sectional	Saudi Arabia	IBD-DISK	IBD	80	18.8	55.0	32.5 ± 11.9	5.7 ± 3.9	45.0	77
Morreale^38^	Cross-sectional	Italy	IBD-DI	UC	37	51.4	–	46	–	–	10
Nardone^14^	Cross-sectional	Italy	IBD-DISK	IBD	767	38.3	49.0	42 (30-57)	15 (6-40)	55.0	68.5
Reves^39^	Cross-sectional	United States- Europe	IBD-DI	IBD	224	29.9	–	35 (25-49)	–	–	40
Shafer^25^	Cross-sectional	Canada	IBD-DI	IBD	838	43.9	63.2	–	–	–	–
				CD	439	47.8	63.3				
				UC	399	40.9	62.2				
Tannoury^40^	Cross-sectional	France	IBD-DISK	IBD	1700	46.7	53.0	–	13.1 ± 10.3	56.0	75
Van Der Have^41^	Cross-sectional	Netherlands	IBD-DI	IBD	1108	7.0	–	–	–	–	13.3
Williet^42^	Cross-sectional	France	IBD-DI	IBD	1183	33.8	61.6	45 (34-60)	14 (7-22)	58.4	26.9
				CD	721	34.7	–	44 (34-58)		51.3	29,8
				UC	462	32.5	–	47 (36-61)		69.5	22,3
Yzet^43^	Cross-sectional	France	IBD-DISK	CD	85	28.2	51.8	38 (33-44)	12 (5-20).	–	–

Abbreviations: CD, Crohn disease; IBD, inflammatory bowel disease; UC, ulcerative colitis.

Risk of bias, measured using the JBI, is summarized in Figure S1 and shows that among the 17 included studies, 12 studies^14^^,^^20^^,^^21^^,^^34–39^^,^^41^^,^^42^ were rated as having moderate methodological quality, while 6 studies^7^^,^^19^^,^^25^^,^^33^^,^^40^^,^^43^ were of high quality.

### Pooled prevalence of moderate-to-severe disability in IBD, CD, and UC

**Figure 2:**
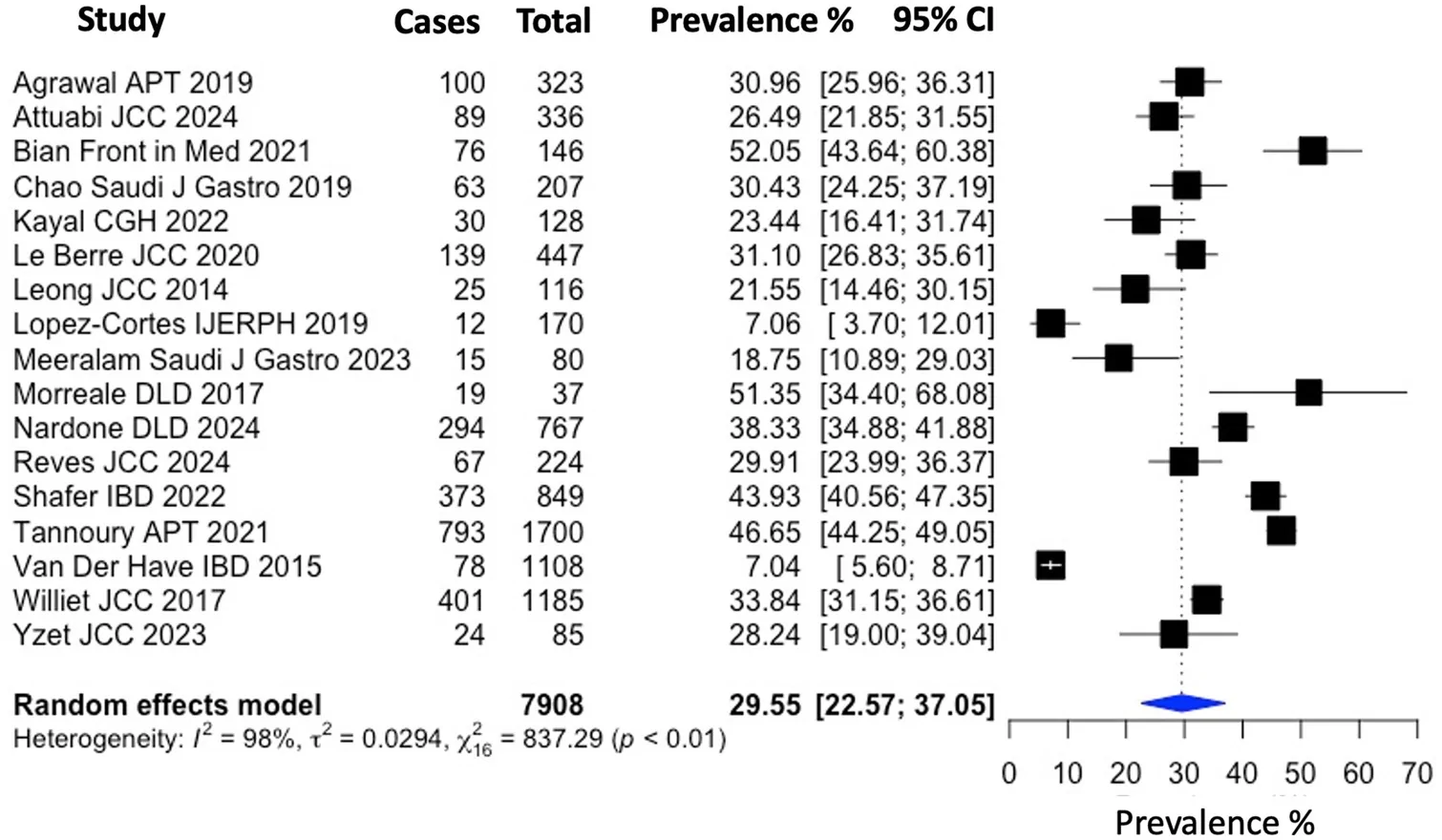
Pooled prevalence of moderate to severe disability in patients with inflammatory bowel disease (IBD).

Based on 17 studies including 7897 patients,^7^^,^^14^^,^^19–21^^,^^25^^,^^33–35^^,^^37–43^ the pooled prevalence of moderate-to-severe disability in patients with IBD was 29.6% (95% CI, 22.6%-37.1%; *I*^2^ = 98.0%, *P* < .01) (Table 1, Figure 2) with no evidence of funnel plot asymmetry (Egger test, *P* = .10), suggesting a low risk of publication bias influencing the pooled estimate (Figure S2).

When we considered CD and UC separately, the pooled prevalence of moderate-to-severe disability in CD was 36.9% (95% CI, 25.7%-48.9%; *I*^2 = ^89%, *P* < .01) based on 6 studies comprising 1594 patients,^21^^,^^25^^,^^33^^,^^36^^,^^42^^,^^43^ and 30.8% (95% CI, 19.6%-43.2%; I^2 = ^86.0%, *P* < .01) in 6 studies including 1275 patients with UC^21^^,^^25^^,^^33^^,^^36^^,^^42^^,^^43^ (Table 1, Figures S3 and S4). In the 4 studies ^25^^,^^33^^,^^36^^,^^42^ that reported data for both IBD types, prevalence was higher in patients with CD compared to UC (OR 1.26, 95% CI, 1.06-1.51), with low levels of heterogeneity between studies (*I*^2^ = 4.6%; *P* = .37) (Figure S5).

### Prevalence of moderate-to-severe disability across countries

**Figure 3:**
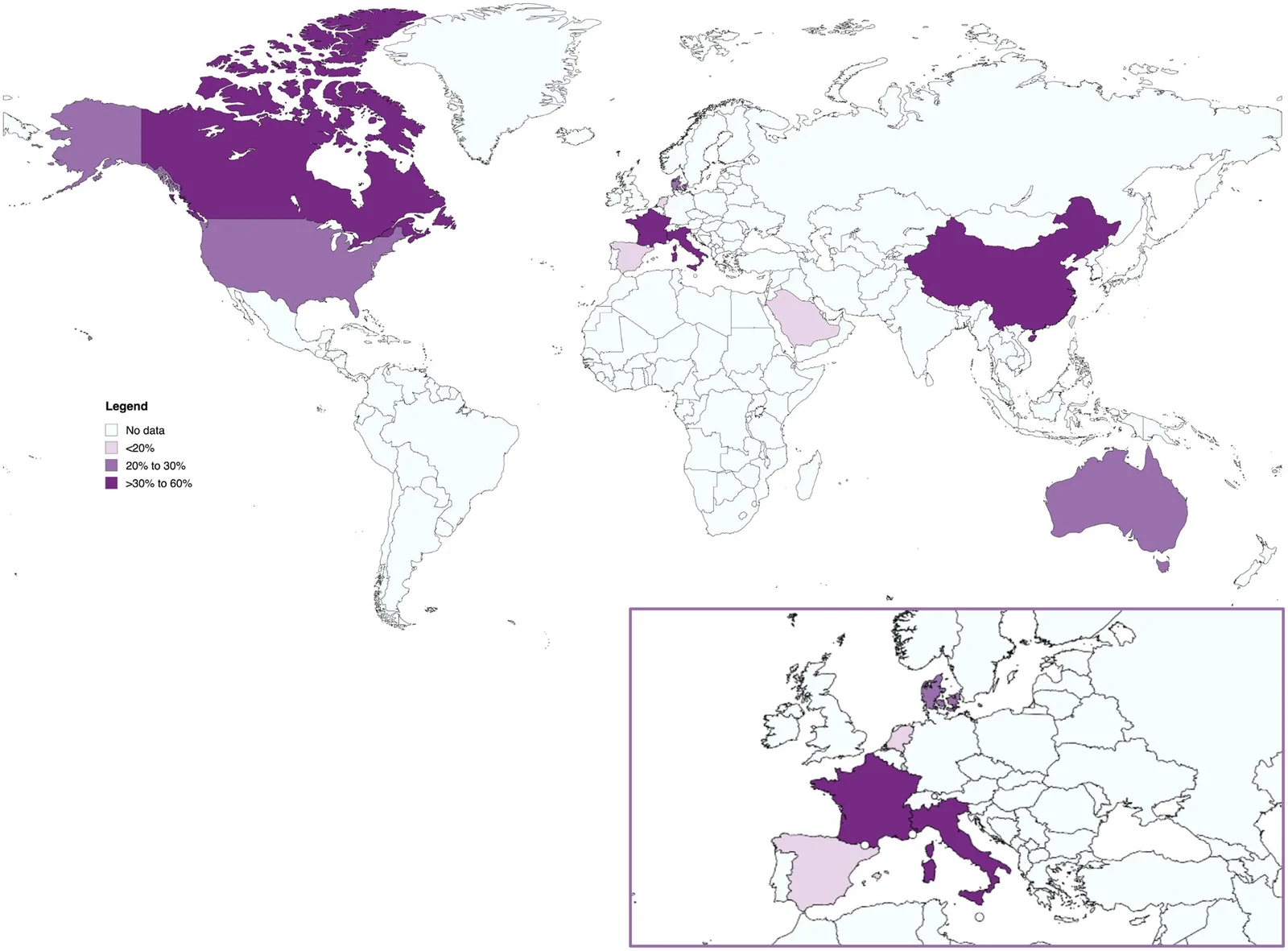
Global prevalence of moderate-to-severe disability in patients with inflammatory bowel disease (IBD).

The pooled prevalence of moderate-to-severe disability varied substantially across countries (Figure 3). The highest prevalence were observed in China,^21^ Italy,^14^^,^^38^ France,^19^^,^^40^^,^^42^^,^^43^ and Canada^25^^,^^34^ where moderate-to-severe disability was reported by 52.0% (95% CI, 44.%-60.1%), 42.4% (95% CI, 30.9%-54.3%, *I*^2^ = 59.1%, *P* = .12), 35.4% (95% CI, 26.9%-44.4%, *I*^2^ = 95.7%, *P* < .001) and 37.4% (95% 24.9%-50.9%, *I*^2^ = 92.3%, *P* < .001) of subjects, respectively. Intermediate rates of moderate-to-severe disability were found in the United States,^7^^,^^35^ Denmark,^33^ Australia^36^ and Saudi Arabia^20^ with 27.9% (95% 21.0%-35.3%, *I*^2^ = 60.5%, *P* = .11), 26.6% (95% CI, 22.0%-31.4%), 21.6% (95% CI, 14.5%-30.1%) and 19.1% (95% CI, 11.3%-28.4%), respectively. Lower rates of moderate-to-severe disability were observed in Spain^37^ (7.3%, 95% CI, 3.9%-11.7%) and in the Netherlands (7.1%, 95% CI, 5.6%-8.7%).^41^

Finally, we observed no significant correlation between GDP per capita and prevalence of moderate-to-severe disability across countries (*r* = −0.26; 95% CI, −0.66-0.25).

### Prevalence of moderate-to-severe disability according to disease activity

The pooled prevalence of moderate-to-severe disability was higher in patients with active IBD (56.9%, 95% CI, 20.3%-89.9%, *I*^2^ = 93.0%, *P* < .01) compared with those with inactive disease (27.0%, 95% CI, 3.3%-62.0%, *I*^2^ = 93.0%, *P* < .01), based on results from 4 studies (Table 2)^14^^,^^20^^,^^21^^,^^40^ that reported prevalence in both active and inactive disease within the same study population. On the basis of these,^14^^,^^20^^,^^21^^,^^40^ the OR for moderate-to-severe disability in active versus inactive disease was 3.13 (95% CI, 1.74-5.64) with high levels of heterogeneity between studies (I^2^ = 81,4%, *P* < .001).

**Table 2:** Pooled prevalence of moderate-to-severe disability in all patients with IBD, UC and CD and in patients with active or inactive IBD, and pooled prevalence of moderate-to-severe disability based on the type of questionnaire used.

	No. of studies	No. of patients	Pooled prevalence (%)	95% CI	*I* ^2^	*P* value for χ^2^
IBD	17	7897	29.6	22.6-37.1	98.0	<.01
CD	6	1594	36.9	25.7-48.9	89.0	<.01
UC	6	1275	30.8	19.6%-43.2	86.0	<.01
IBD active	4	1436	56.9	20.3-89.7	93.0	<.001
IBD inactive	4	1259	27.0	3.3-62.0	93.0	<.001
IBD-DI	12	4818	28.3	19.1-38.6	98.0	<.001
IBD-DISK	5	3079	33.3	21.1-46.9	94.0	<.001

Abbreviations: CD, Crohn disease; IBD, Inflammatory bowel disease; IBD-DI, IBD Disability Index; IBD-DISK, self-administered, visually appealing, simplified version of the IBD-DI; UC, ulcerative colitis.

### Prevalence of moderate-to-severe disability according to the questionnaire used

All the studies used the IBD-DISK or IBD-DI questionnaires (Table 2).^14^^,^^19^^,^^20^^,^^40^^,^^43^ The prevalence of moderate-to-severe disability measured with IBD-DISK, in 5 studies^14^^,^^19^^,^^20^^,^^40^^,^^43^ was 33.33% (95% CI, 21.06-46.85%, *I*^2^ = 94%, *P* < .001—Figure S6), while in the remainder 12 studies that used the IBD-DI score prevalence^7^^,^^21^^,^^25^^,^^33–39^^,^^41^^,^^42^ was 28.33% (95% CI, 19.05-38.62, *I*^2^ = 98%, *P* < .001—Figure S7) with no evidence of funnel plot asymmetry (Egger test, *P* = .58), suggesting a low risk of publication bias influencing the pooled estimate (Figure S8).

## Discussion

Inflammatory bowel disease (IBD) places a considerable burden on patients, not only through its physical symptoms but also by contributing to long-term disability.^44^ Although the relevance of disability in IBD is increasingly acknowledged, no study to date has systematically assessed this burden on a global scale, as has been done for other chronic conditions. Moreover, prevalence estimates of IBD-related disability have, so far, been fragmented and geographically inconsistent.^45^^,^^46^ To address this gap, we conducted what is to our knowledge the first systematic review and meta-analysis to comprehensively assess the prevalence of moderate-to-severe disability in the IBD population. Moderate-to-severe disability was estimated to affect nearly 1 in 3 patients with IBD, with a greater pooled prevalence in patients with CD than in those with UC.

As expected, the prevalence was considerably higher among patients with active disease compared to those in remission, corresponding to a 3-fold increased odds of moderate-to-severe disability. More surprisingly was the relatively high prevalence of moderate-to-severe disability in patients in remission, which underscores the lasting impact of disease on individual wellbeing even beyond short term control of inflammation.

The highest prevalence rates of moderate-to-severe disability were reported in China,^21^ Italy,^14^^,^^38^ France,^19^^,^^40^^,^^42^^,^^43^ and Canada,^25^^,^^34^ exceeding 35%, suggesting a significant disease burden in these regions. Intermediate prevalence rates were observed in the United States,^7^^,^^35^ Denmark,^33^ Australia,^36^ and Saudi Arabia,^20^ while notably lower rates were seen in Spain^37^ and the Netherlands.^41^ The wide variation in the pooled prevalence of moderate-to-severe disability across countries may partially reflect the progression of IBD through different epidemiological stages.^3^ Variation in environmental exposures and health care systems may further influence the magnitude of these differences. However, we could not identify a common factor plausibly linking regional differences with differences in the prevalence of disability. These variations are more likely due to heterogeneity in study populations than true geographic variation. Yet, all studies included in our meta-analysis employed validated instruments, ensuring formal consistency across countries. Contextual factors may better explain at least some of these differences: in Spain,^37^ over half of participants enrolled were in clinical remission, possibly lowering disability scores, while the Dutch^41^ study used a web-based self-reported version of IBD-DI, a method that may limit accuracy and omit important disease activity markers, introducing potential sampling bias. However, whether disability estimates can vary across different health care contexts has yet to be determined.

Individual variability in socioeconomic factors may also account for a greater share of the observed differences in disability prevalence. Shafer et al. compared the distributional and predictive properties of the IBD-DI across 5 cities in 3 different countries—Winnipeg, Chicago, Toronto, Hong Kong, and Jerusalem—and showed that the severity of disability based on IBD-DI score was directly comparable across these settings despite differing in terms of culture and government support.^22^ However, all study sites were located in economically developed regions with relatively similar health care standards. As such, the generalizability of these findings to under-resourced or structurally different health care systems is limited. In our study no significant correlation was found between per capita GDP and the reported prevalence of moderate-to-severe disability. We acknowledge that this analysis was exploratory and limited by the small number of countries included (*n* = 17). In addition, GDP per capita may also be a poor proxy for health care access or social support systems. Therefore, any interpretation of the lack of correlation should be made cautiously.

Conversely, in a multicenter, cross-sectional study conducted across 5 academic institutions in New York City,^7^ racial and ethnic minority individuals, public insurance coverage, and low household income were each associated with a 2-to-3–fold higher likelihood of reporting moderate-to-severe disability among patients with IBD, independent of disease-related factors. Similarly, previous studies have reported that unemployment is associated with higher IBD-DI scores, and lower levels of education correlate with reduced quality of life.^42^^,^^47^^,^^48^ These findings underscore the influence of socioeconomic and cultural environments on patient lived experience of disability and point to inequities in health care access among vulnerable groups.

Consistent with previous studies,^40^^,^^49^^,^^50^ in our study disease activity remains a key driver of IBD-related disability and is closely linked to adverse outcomes. The pooled prevalence of moderate-to-severe disability was higher in patients with IBD with active disease, with 3-fold higher odds.^14^^,^^20^^,^^21^^,^^40^ Indeed, symptoms associated with active disease—including abdominal pain, diarrhea, rectal bleeding, and extraintestinal manifestations—substantially affect physical and mental functioning, with consequent limitations in participation in daily life activities. In addition, individuals with active disease at baseline and no previous history of mental disorders exhibit a higher risk of developing anxiety or depression over time,^51^ with consequent adverse effects on disease acceptance, coping strategies, and engagement in care.^52^ Thus, disease activity triggers a cascade of consequences, including psychological distress, work impairment, emotional and interpersonal dysfunction, and sexual difficulties, that together encompass the multidimensional concept of disability in IBD.

We further reported a higher pooled prevalence of moderate-to-severe disability in CD than in UC. Multiple international studies consistently show that CD is associated with greater disability than UC. In a multinational cohort of 1972 patients, CD was linked to a 1.3-fold higher risk of severe disability.^22^ A French and Belgian study also found lower odds of moderate-to severe disability in patients with UC compared to those with CD.^40^ In the Netherlands,^41^ CD patients had significantly worse functional scores and more frequent complaints of abdominal pain, bowel control, self-care, community engagement, interpersonal relationships, and work or household responsibilities. These factors likely account for the higher disability burden observed in CD.

Finally, we investigated the prevalence of moderate-to-severe disability based on the questionnaire used. Using the IBD-DISK, the pooled prevalence of MSD was 33.3%, whereas in studies using the IBD-DI, it was slightly lower at 28.3%. The similar estimates between IBD-DISK and IBD-DI confirm the validity of both tools in capturing functional impairment, although differences in structure and administration may influence reporting. Indeed, the IBD-DI is a physician-administered tool, primarily used in clinical trial settings. Of note, its non–self-administered format may lead to discrepancies between the patient’s lived experience and the physician’s interpretation. In contrast, the IBD-Disk is a self-administered, user-friendly tool that can be implemented easily in routine clinical practice, such as in outpatient waiting rooms or infusion units.^53^ However, to date, no direct comparison between the IBD-Disk and the IBD-DI has been conducted. Notably, no included study directly compared moderate-to-severe disability measured with IBD-DI and IBD-Disk in the same patient cohort. Therefore, any interpretation regarding the comparability of the 2 tools should be considered only as hypothesis generating.

The main strength of this study is that it used validated questionnaires to provide the first estimate of the pooled prevalence of disability in IBD across 17 countries . Although we observed variability in disability prevalence across studies, there was no clear association between these differences and the economic metrics of the countries in which the studies were conducted. This finding suggests that the heterogeneity we observed may instead stem from differences in the populations recruited, such as clinical characteristics, disease activity, or health care access, rather than from broader socioeconomic factors. Furthermore, by demonstrating a substantially higher burden of disability among patients with active IBD, our findings underscore the value of incorporating disability assessment into both clinical decision-making and routine disease monitoring, especially in the context of a treat-to-target approach or in disease modification trials.^54^ In this regard, the use of disability tools at baseline before starting a new therapy and during follow-up may serve as a valuable noninvasive adjunct for targeting interventions. Nevertheless, we found that more than 25% of patients in clinical remission still reported symptoms related to disability. The reasons for this might be various, including the accumulated damage to the gastrointestinal tract, the high prevalence of other immune-mediated conditions, the psychological burden of requiring long-term medical treatment or surgery, and, last but not least, alterations in the brain-gut axis. These proposed mechanisms are speculative and were not directly evaluated in the included studies. We therefore present them as hypothesis-generating considerations for future research. Given the close interplay between the brain and gut, it is important to assess disability even in patients who are in remission because psychological symptoms may increase the risk of future flares of IBD activity.^51^^,^^55^ Consistent with these findings, a recent meta-analysis^51^ identified depression and anxiety as strong determinants of adverse clinical outcomes in IBD, including higher rates of hospitalization, emergency department utilization, treatment escalation, and surgery.

Limitations of the study should be acknowledged. Specifically, data on the prevalence of moderate-to-severe disability were available for only 17 countries. This prevents a comprehensive understanding of the global distribution and determinants of disability across different regions, particularly in those with lower economic income. The lack of country-level data on health-care resources and access to high-cost therapies further precluded assessment of whether differences in treatment accessibility contribute to variations in disability.

In addition, the majority of included studies were cross-sectional and conducted in tertiary care settings, which may have introduced selection bias and led to overestimation of disability rates due to overrepresentation of more severe or complex cases. Nevertheless, the pooled prevalences from multiple studies provide a general overview of the prevalence of moderate-to-severe disability and enhance the generalizability of our findings. Furthermore, we could not analyze the differences in prevalence of moderate-to-severe disability according to sex, educational attainment, race, or income. We did not assess the prevalence of moderate-to-severe disability based on age, because most studies did not report the mean age of included individuals according to disability status. Notably, in all included studies, the median age ranged from 31 to 47 years, reflecting that participants in clinical studies are predominantly younger adults, with populations aged >60 years underrepresented.

Further research is needed to explore disability in specific subgroups of patients such as the older adults and the correlation of disability with socioeconomic factors and should incorporate broader geographic representation, along with greater attention to sex- and ethnicity-related insights worldwide.

Finally, the lack of stratification by disability level for key disease characteristics—including duration, location, behavior, and therapeutic exposure—limited quantitative data extraction and comparative analysis. Nonetheless, evidence from large cohort studies suggests that disease duration and phenotype are not independently associated with disability.^40^^,^^41^^,^^56^ Consistent with this evidence, preliminary findings showed no association between disability assessed by the IBD-DI and cumulative bowel damage quantified using the Lémann index, a measure of long-term structural damage.^39^ The association between biologic therapy and disability remains heterogeneous across studies: while some reports describe higher disability rates among patients treated with anti-TNF agents,^22^^,^^57^ others do not report observations of independent associations.^40^^,^^41^ Overall, these data supported the view that disability is largely driven by active disease burden rather than by disease phenotype or therapeutic class per se.

Overall, our study underscores the significant burden of moderate-to-severe disability among patients with IBD, with implications for clinical management and the importance of country-specific considerations in understanding and addressing equity of care. The high prevalence of moderate-to-severe disability highlights its impact on patients with IBD, a global health concern that crosses national borders and calls for coordinated international strategies.^58^ As the prevalence of IBD continues to increase globally, the burden of disease risks is deepening existing health inequities and placing unsustainable pressure on health care systems. Although current models of care are increasingly patient centered, this approach must be accompanied by greater attention to the social determinants of health. Social disparities can limit an individual’s ability to participate fully in daily life, effects that contribute to IBD-related disability and poorer outcomes. Addressing such manifestations of disability requires a dual commitment: tailoring care to individual needs, while also engaging with national and international stakeholders to reduce the environmental and social impact of disability and ensure equitable access to care for all patients.

In conclusion, this meta-analysis of 17 studies from 17 countries shows that nearly one-third of patients with IBD live with moderate-to-severe disability. While prevalence varied markedly across studies, no consistent geographic patterns emerged. Moderate-to-severe disability was more common among individuals with CD and those with active inflammation, highlighting the strong association between disease activity and functional impairment. Notably, even among patients in clinical remission, the prevalence of moderate-to-severe disability remained high at 27%, underscoring the persistence of disability beyond overt disease activity. These findings reinforce the importance of systematically assessing disability in clinical practice and research. However, further longitudinal research is needed to clarify its role in guiding therapeutic decisions.

## Supplementary Material

izag022_Supplementary_Data

## Data Availability

The data underlying this article will be shared on reasonable request to the corresponding author.
