# Supplementary figures and images for: Prevalence of disability in inflammatory bowel disease: a systematic review and meta-analysis
Source: Inflamm Bowel Dis. 2026 Mar 5;32(7):1256–67. doi: 10.1093/ibd/izag022 (PMC13337221; doi:10.1093/ibd/izag022)

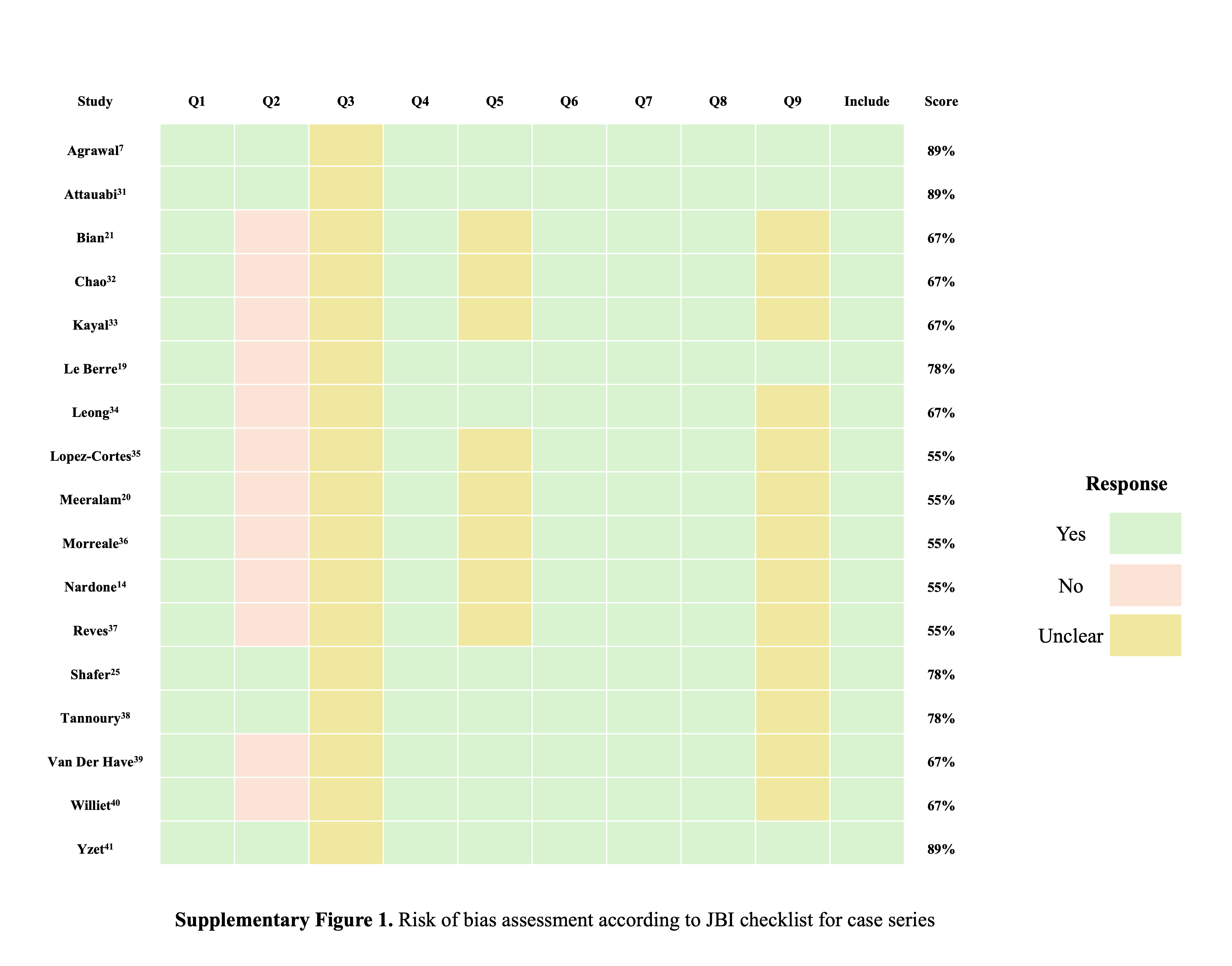

Supplement: izag022_Supplementary_Data [file izag022_supplementary_data.zip › Supplementary Figure 1.tiff]

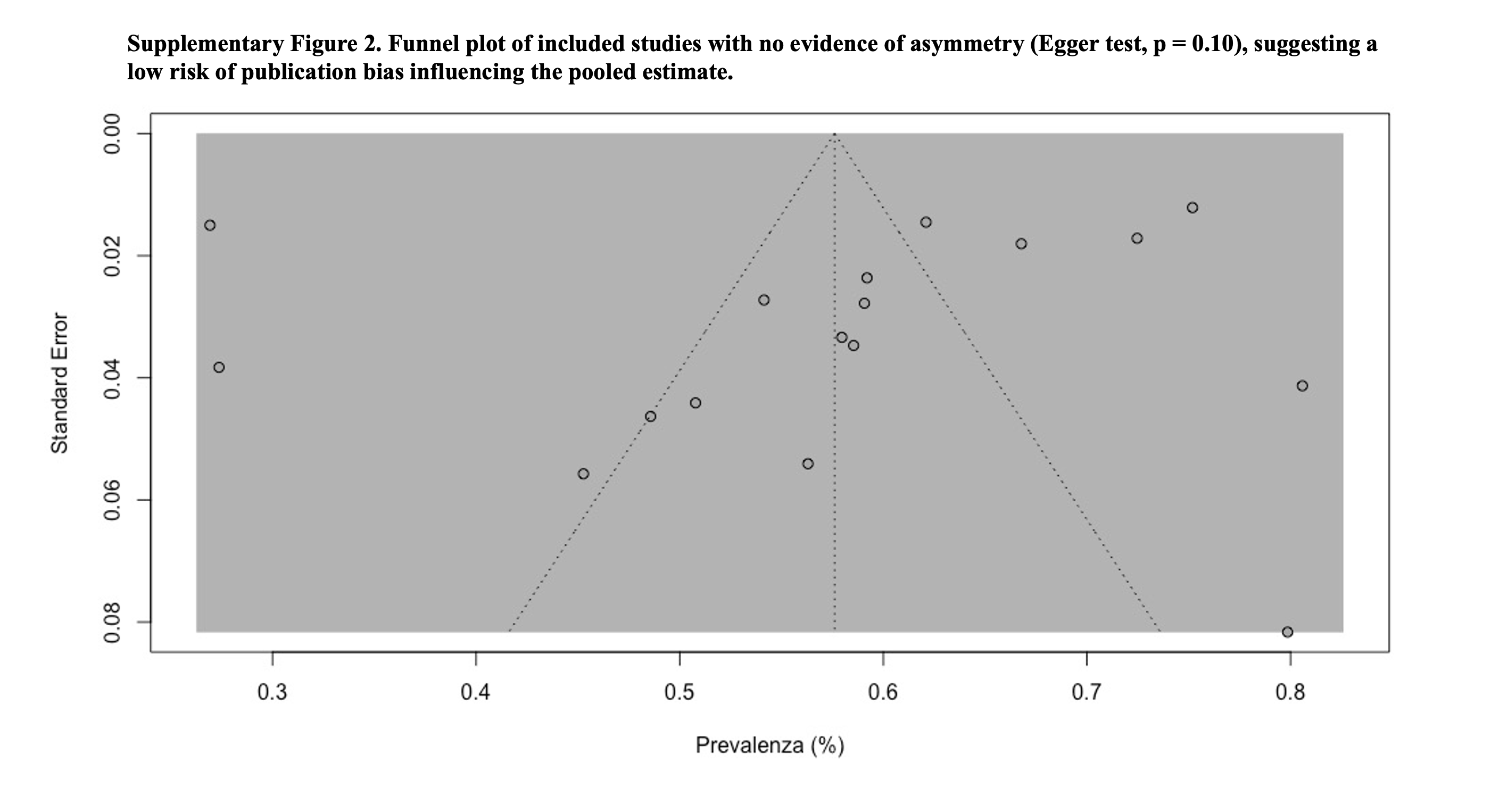

Supplement: izag022_Supplementary_Data [file izag022_supplementary_data.zip › Supplementary Figure 2.tiff]

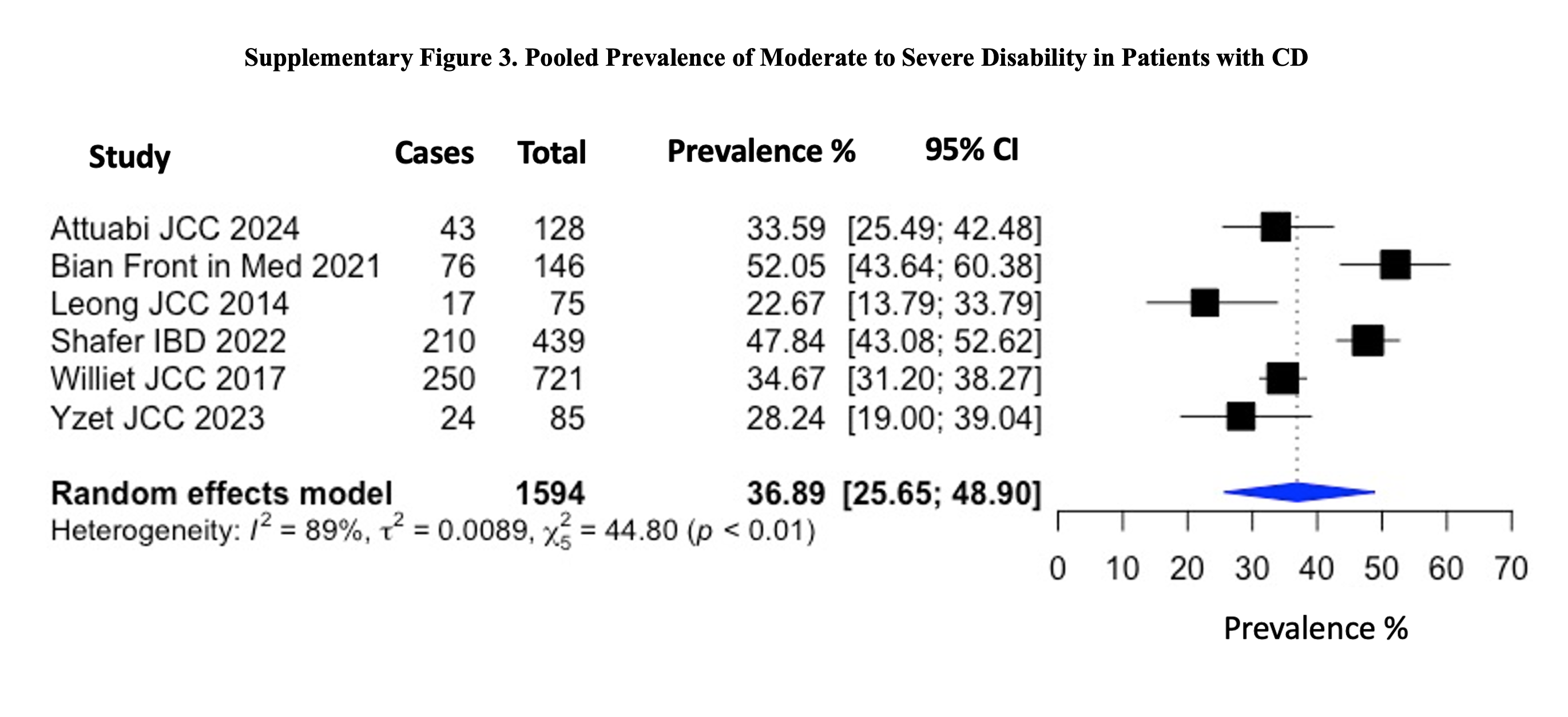

Supplement: izag022_Supplementary_Data [file izag022_supplementary_data.zip › Supplementary Figure 3.tiff]

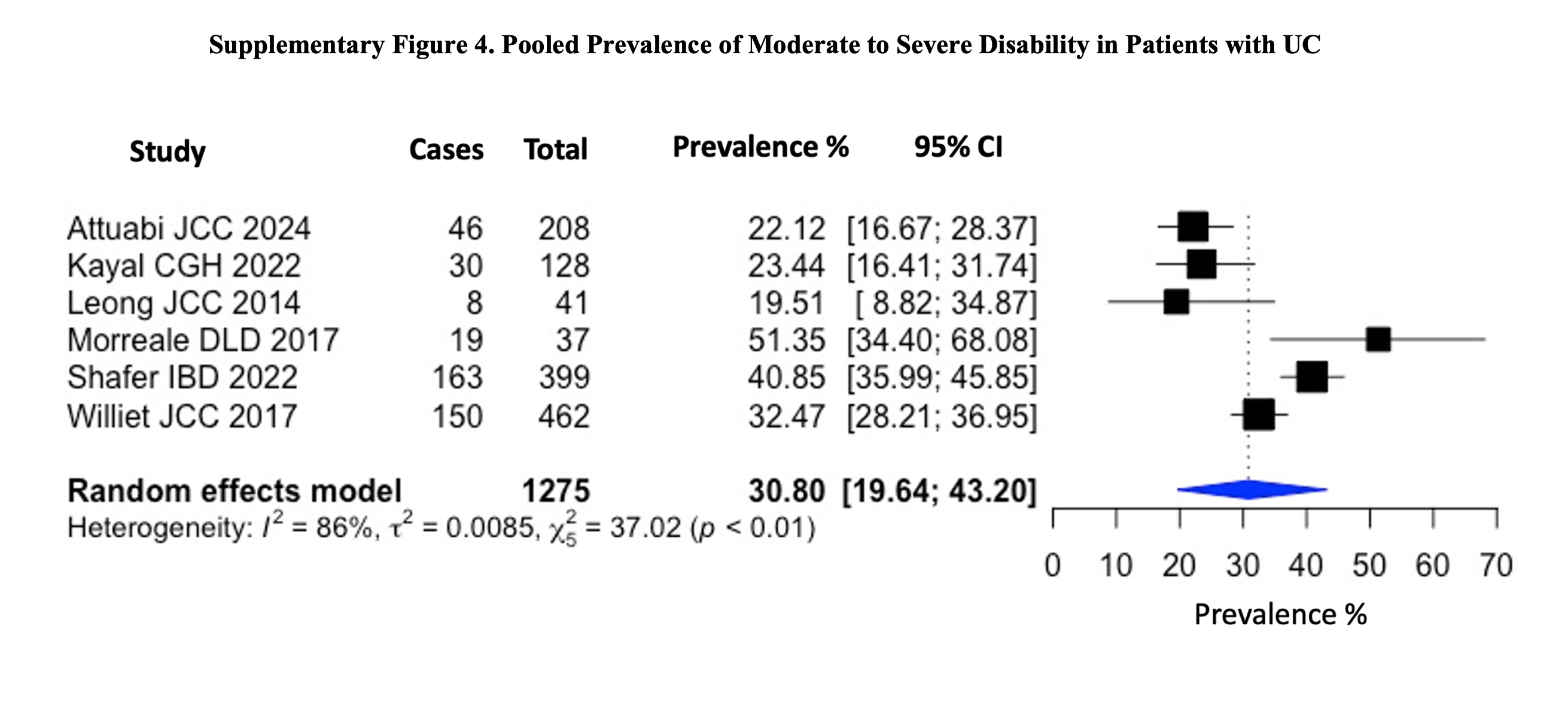

Supplement: izag022_Supplementary_Data [file izag022_supplementary_data.zip › Supplementary Figure 4.tiff]

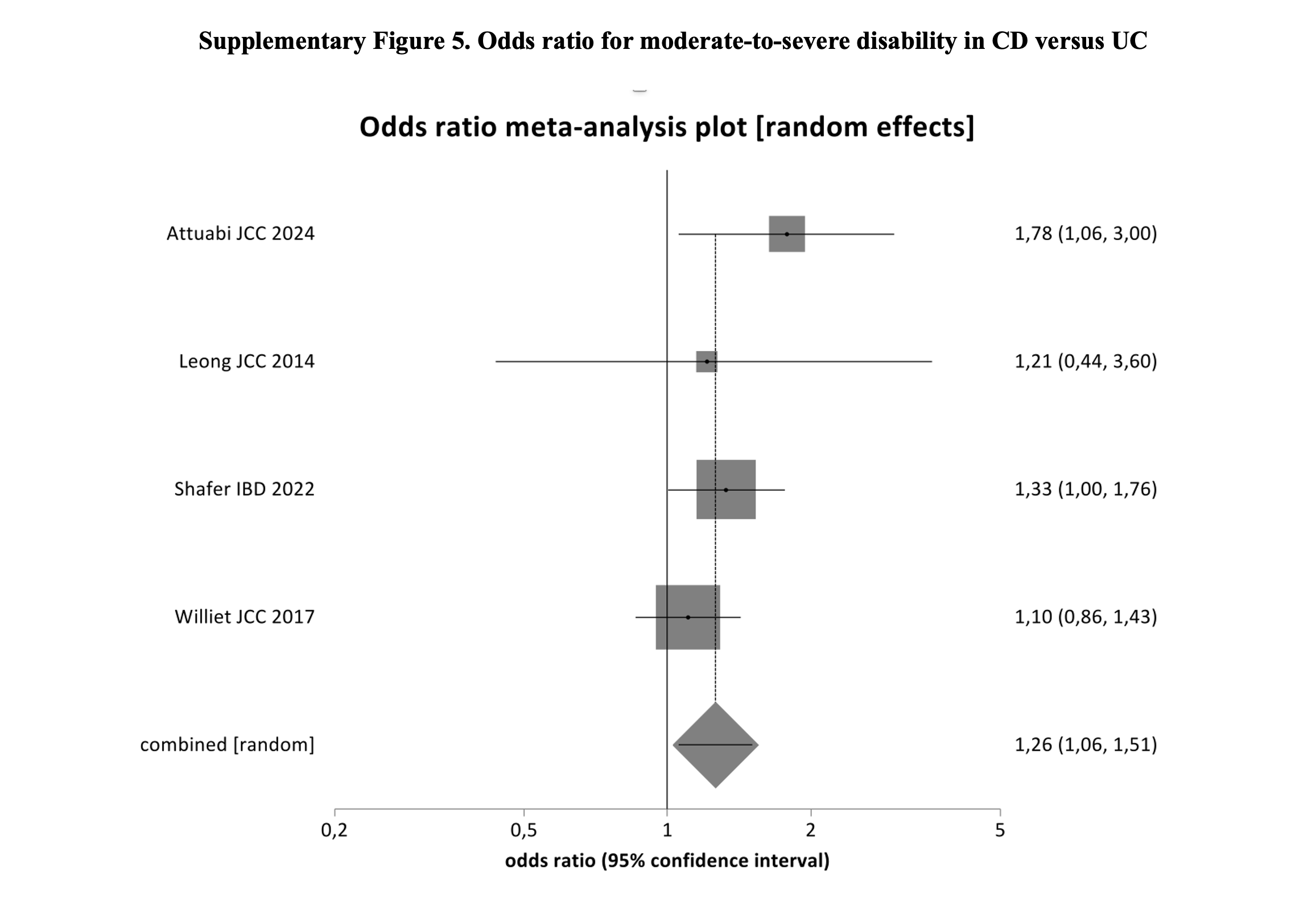

Supplement: izag022_Supplementary_Data [file izag022_supplementary_data.zip › Supplementary Figure 5.tiff]

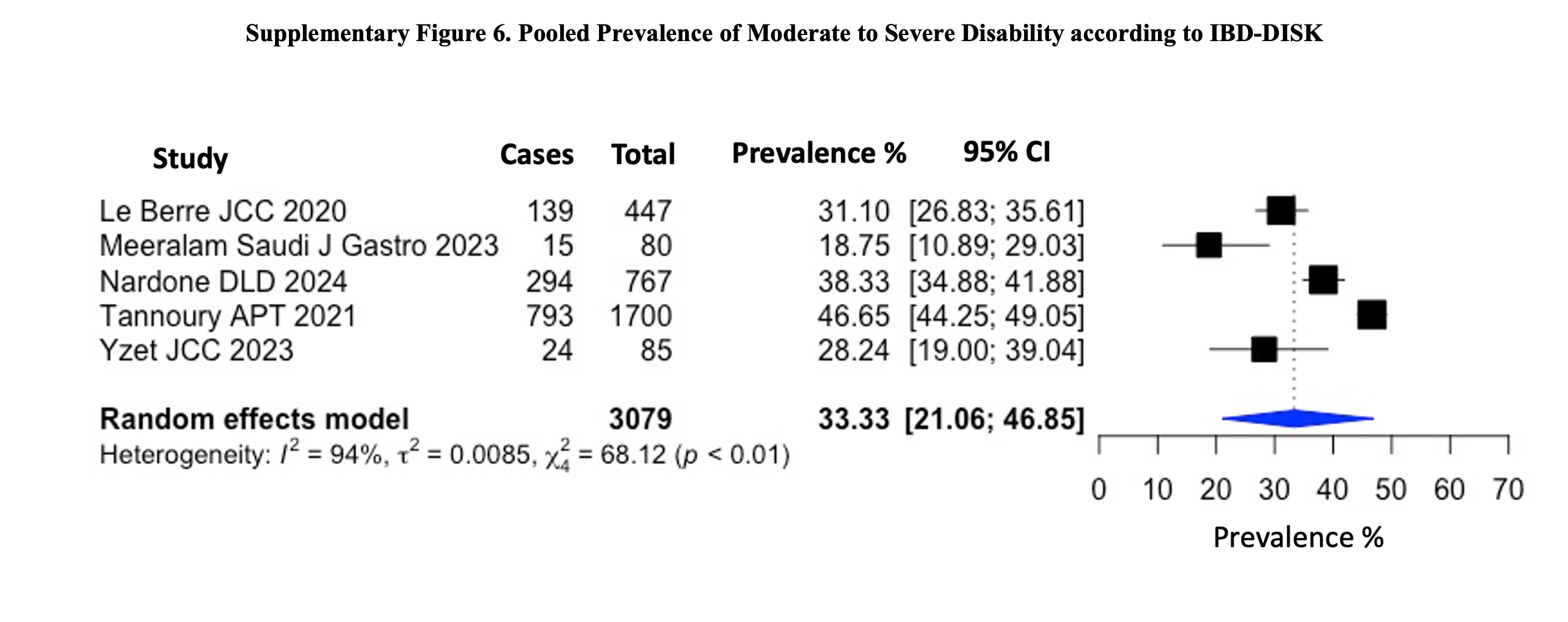

Supplement: izag022_Supplementary_Data [file izag022_supplementary_data.zip › Supplementary Figure 6.tiff]

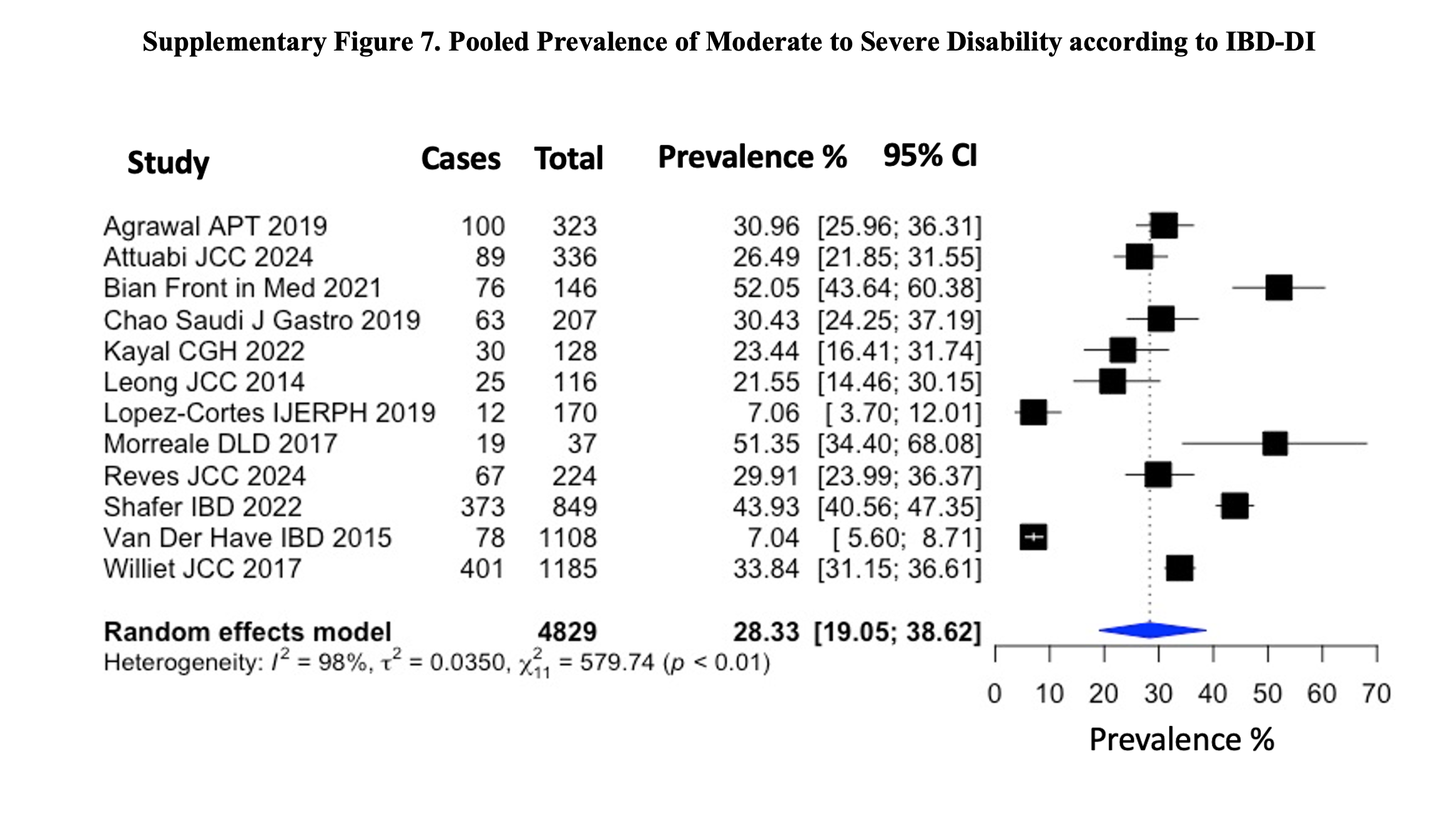

Supplement: izag022_Supplementary_Data [file izag022_supplementary_data.zip › Supplementary Figure 7.tiff]

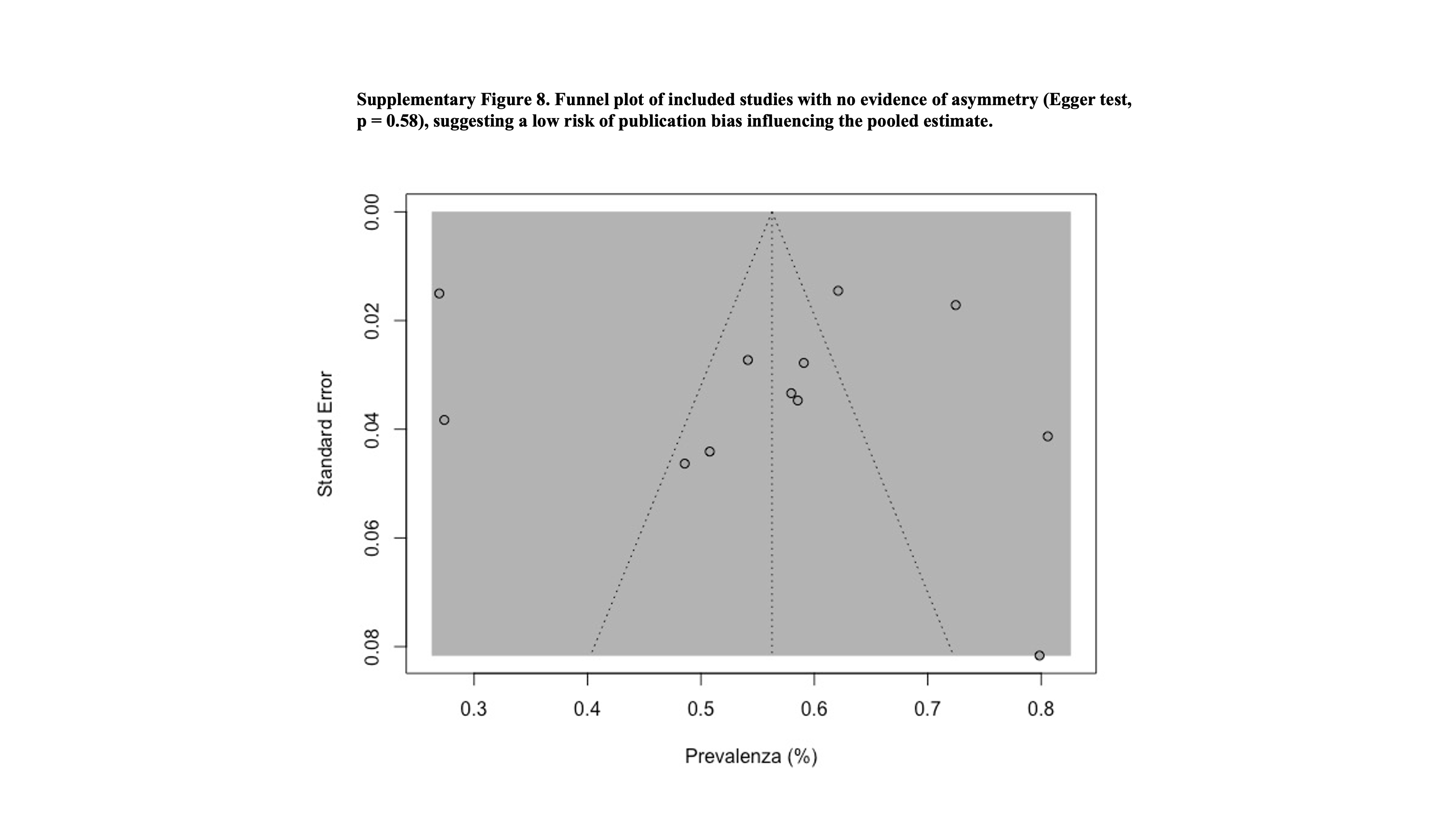

Supplement: izag022_Supplementary_Data [file izag022_supplementary_data.zip › Supplementary Figure 8.tiff]
